# Association between air pollution and non-accidental mortality in Guiyang, China: a time-series analysis (2013–2023)

**DOI:** 10.3389/fpubh.2025.1602900

**Published:** 2025-07-16

**Authors:** Xuanhao Chen, Minmin Su, Minlan Yuan, Zihai Jian, Dan Yang, Ruifang Liu, Hua Guo, Jianhua Zhang

**Affiliations:** Guizhou Center For Disease Control And Prevention, Guizhou, China

**Keywords:** air pollutants, daily mortality, excess risks, time-stratified case-crossover design, generalized additive model (GAM)

## Abstract

**Background:**

Air pollution remains one of the leading environmental risk factors for human health globally, significantly contributing to the burden of disease and premature mortality. The relationship between air pollutants and non-accidental deaths in Guiyang was unclear.

**Method:**

Descriptive analysis was conducted to characterize air pollutants and mortality trends during the study period. A time-stratified case-crossover design and a quasi-Poisson regression within a generalized additive model (GAM) framework were employed to examine the association between air pollution and mortality. The models incorporated a 7-day lag structure and adjusted for temporal trends, meteorological factors, and day of the week. Analyses were further stratified by cause-specific mortality, age group, sex, and seasonaty as an endpoint for acute health effects, stratified by cause-specific mortality, age, sex and season.

**Results:**

SO₂ exposure was associated with a 3.18% (95% CI: 1.95 to 4.42%) increase in excess risk (ER) for non-accidental mortality per 10 μg/m^3^ increase, with risks peaking at lag 1 and cumulative effects persisting beyond 7 days. NO₂ exposure resulted in a 2.87% (95% CI: 1.72 to 4.03%) ER increase, peaking at lag 1, with effects extending to lag 7. PM₂.₅ and PM₁₀ exposure resulted in ER increases of 1.42% (95% CI: 0.84 to 1.99%) and 0.86% (95% CI: 0.50 to 1.22%), respectively, peaking within 1–2 days. CO exposure was associated with a 1.64% (95% CI: 1.01 to 2.29%) ER increase per 0.1 mg/m^3^, with effects observed at lag 0. Females showed higher susceptibility to SO₂ (ER: 3.87, 95% CI: 2.05 to 5.72%), while males were more vulnerable to PM₂.₅ (ER: 1.80, 95% CI: 1.10 to 2.51%). Individuals aged ≥65 years exhibited elevated risks across all pollutants, particularly SO₂ (ER: 4.16, 95% CI: 2.73 to 5.61%). All pollutants contributed to excess mortality in the cold season.

**Conclusion:**

Different pollutants exhibit different lag durations of excess risks and cumulative excess risks for non-accidental mortality and deaths related to circulatory and respiratory systems. Females showed greater susceptible to SO_2_, while males were more vulnerable to other pollutants. The individuals aged 65 years and older were particularly vulnerable populations.

## 1 Introduction

Air pollution remains one of the leading environmental risk factors for human health globally, significantly contributing to the burden of disease and premature mortality. According to the World Health Organization (WHO), 99% of the world’s population is exposed to air quality levels that exceed recommended limits. It is estimated that 8.7 million deaths worldwide result from breathing in particulate matter emitted into the atmosphere by fossil fuel combustion (1). In 2019, ambient air pollution was estimated to have caused 4.2 million premature deaths worldwide, primarily affecting cardiovascular and respiratory systems (2, 3). In 2021, particulate matter pollution, including both outdoor and indoor sources, was identified as a leading environmental risk factor for global health, according to the Global Burden of Disease Study (4). Air pollution is closely linked to cardiovascular and respiratory mortality. Fine particulate matter, due to its small particle size, can penetrate deeply into the lungs and bloodstream, causing significant damage to both cardiovascular and respiratory systems (5, 6). Gaseous pollutants like SO_2_ and NO_2_ are more associated with respiratory effects through airway irritation. Vulnerability varies by age and sex, with older adults (≥65 years) showing higher risks due to reduced physiological resilience, and females potentially more susceptible to gaseous pollutants due to greater airway reactivity (7, 8).

Guiyang, the capital of Guizhou Province, is located on the eastern edge of the Yunnan–Guizhou Plateau. The city features mountainous terrain, high forest coverage, and a humid subtropical climate with frequent rainfall, largely influenced by a quasi-stationary front. These unique geographical and meteorological characteristics can influence the generation, dispersion, and health impacts of air pollutants. Although Guiyang generally experiences lower pollution levels than many other Chinese cities, it remains a less-developed urban area with limited environmental management capacity. The uneven distribution of environmental risks across population raises concerns about environmental equity. Most existing studies in China have focused on heavily polluted or economically developed cities (8, 9). Evidence from less-developed inland cities, featuring unique geographic and climatic conditions as well as socioeconomic profiles, remains scarce. This study addresses this gap by investigating the associations between ambient air pollutants and mortality in Guiyang, providing region-specific evidence to inform public health interventions and promote environmental justice in under-resourced settings.

We employed a time-stratified case-crossover design combined with generalized additive models (GAMs) to evaluate the short-term effects of air pollutants on all-cause and cause-specific mortality in Guiyang. We also assessed potential effect modifications by age, sex, season, and cause of death to identify particularly vulnerable subgroups. Localized studies such as this are essential for capturing context-specific exposure–response relationships and informing the development of targeted air quality management strategies aimed at enhancing environmental health protections.

## 2 Materials and methods

### 2.1 Data sources

Mortality data were obtained from the Disease Surveillance Points System, managed by the Chinese Center for Disease Control and Prevention, spanning January 1, 2013, to December 31, 2023. The data included a range of causes of death based on the primary diagnosis coded by ICD-10 (international classification of diseases, 10th revision), including non-accidental causes (A00-R99), respiratory cases (J00-J99), cardiovascular cases (I00-I99). We excluded the data from December 18, 2022, to January 28, 2023, due to the COVID-19 outbreak in Guiyang, which disrupted mortality patterns and caused an abnormal increase in deaths, potentially introducing significant bias into the analysis. Daily temperature and relative humidity data (2013–2023) were sourced from the Guiyang Meteorological Bureau. Daily pollutant concentrations (SO_2_, NO_2_, PM_2.5_, PM_10_, and CO) were calculated as the arithmetic mean across data from 12 air quality monitoring stations in Guiyang’s main urban area, following standard time-series studies. All stations operational during the study period (2013–2023) were included. Missing values for air pollutant data, accounting for less than 1% at each station, were random and sporadic. Given the spatial averaging across multiple monitoring sites, these localized gaps had negligible impact on city-level daily means. Thus, the missing data were excluded from the analysis. Meteorological data had no significant missingness.

### 2.2 Statistics analysis

Descriptive statistics including the 25th percentile (*P*25), median (*P*50), 75th percentile (*P*75), minimum, and maximum were calculated for daily death counts, temperature, relative humidity, and pollutant concentrations (CO, PM_2.5_, PM_10_, SO_2_, and NO_2_). Spearman correlation analysis was applied to examine the relationships between pollutants and meteorological factors. Variance inflation factor (VIF) analysis was used to assess multicollinearity among pollutants.

We adopted a hybrid approach by integrating a GAM-based time-series framework with a time-stratified case-crossover design to assess the association between air pollution and mortality. The case-crossover design, validated in environmental epidemiology research (10). This design compares exposures during case periods (days preceding death) with matched control days (same weekday within the same month), effectively controlling for temporal trends and seasonality while estimating acute effects of air pollutants on mortality (11). Given the 11-year duration of our study, smooth term for time was included in the model to capture smooth, long-term variations over extended periods that case-crossover design may not fully captured (12).

Given that daily death counts relative to the total population represent a low to probability event, a generalized additive model with a quasi-Poisson distribution was employed. The formula is as follows:


$$\begin{array}{l} \mathrm{lg}[E\;(Y_{t})\;]={\mathrm{\beta Z}}_{t}+\mathrm{ns}\;(\text{time},\mathrm{df})+\mathrm{ns}\;(\text{temp},\mathrm{df})+\mathrm{ns}(\text{rhum},\mathrm{df})+\\ \;\;\;\;\;\;\;\;\;\;\;\;\;\;\;\;\;\;\;\;\;\;\;\;\;\;\;\;\;\;\mathrm{Dow}+\text{stratum}+\text{intercept} \end{array}$$

Where *Y_t_* represents number of deaths on day *t*. *β* represents the regression coefficient. *Z*_t_ represents concentration of a single pollutant on day *t*. *ns()* is natural spline smoothing function. *Time* is the variable of the temporal trend.; *df* is degrees of freedom. *Temp* is daily average temperature. *Rhum* is daily average relative humidity; Dow represents day to week effect; Stratum is time stratification variable; Intercept is model intercept.

Degrees of freedom were set to 7 per year for time, 3 for temperature, and 3 for relative humidity, optimized using the minimum quasi Akaike Information Criterion (QAIC).

Lag effects were analyzed for lag 0 to lag 7, and cumulative lag effects were assessed for lag 01 to lag 07. The acute mortality risk from air pollutants, while not always instantaneous, is predominantly concentrated within the first week following exposure (13, 14). The data were stratified into four subgroups: cause-specific mortality (overall, respiratory and cardiovascular), age (<65 years and ≥65 years), a stratification used to define older adults with greater vulnerability to air pollution and consistent with a large body of literature, sex (male and female) and season (defined October to March as cold season and April to September as warm season). Effects were considered statistically significant if the 95% confidence interval (CI) excluded zero. The analyses were performed primarily using the *Hmisc* and *mgcv* packages in R software (version 4.3.1). The significance level was set at *α* = 0.05.

### 2.3 Sensitivity analysis

To test the robustness of our findings, we conducted sensitivity analyses by varying the degrees of freedom (df) for the time trend (6–8 per year), temperature (3–6), and humidity (3–6). We also fitted two-pollutant models, adjusting for one co-pollutant at a time, to assess potential confounding.

Furthermore, we evaluated the temporal stability of the association by stratifying the analysis into three periods: a baseline (2013–2019), the full period (2013–2023), and an adjusted period which excluded the acute COVID-19 outbreak phase. This comparison allowed us to assess the consistency of the effect estimates.

## 3 Results

### 3.1 Descriptive statistics

From 2013 to 2023, a total of 140,099 non-accidental deaths were recorded in Guiyang’s main urban area, comprising 83,272 males (59.44%), and 56,827 females (40.56%). Deaths due to circulatory and respiratory system diseases totaled 59,830 and 21,028, respectively (Table 1). The average temperature and relative humidity were 15.3°C and 80.3%, respectively. Daily average concentrations of CO, NO_2_, SO_2_, PM_2.5_, and PM_10_ were 0.60 μg/m^3^, 22.5 μg/m^3^, 13.8 μg/m^3^, 32.4 μg/m^3^ and 53.1 μg/m^3^, respectively (Table 2). The results for the entire study period are presented in Supplementary Tables S1, S2.

**Table 1:** Distributions of non-accidental mortality in Guiyang, 2013–2023.

Variables	*n*	x ± s	Min	*P*25	P50	P75	Max
Number of non-accidental deaths	140,099	32.3 ± 16.2	1.0	21.0	37.0	48.0	100.0
Number of respiratory system deaths	21,028	5.3 ± 3.3	0.0	3.0	5.0	7.0	26.0
Number of circulatory system deaths	59,830	15.1 ± 7.7	0.0	9.0	15.0	20.5	45.0
Male	83,272	21.0 ± 9.9	0.0	13.0	21.0	28.0	58.0
Female	56,827	14.3 ± 7.5	0.0	8.0	14.0	20.0	42.0
Age 0–64 years	37,460	9.4 ± 4.8	0.0	6.0	9.0	13.0	28.0
Age ≥65 years	102,639	25.8 ± 12.7	0.0	15.0	26.0	36.0	73.0

Data exclude the period from December 18, 2022, to January 28, 2023, due to the COVID-19 outbreak.

**Table 2:** Daily air pollutants concentration and meteorological factors in Guiyang, 2013–2023.

Variables	x ± s	Min	*P*25	*P*50	*P*75	Max
Temp (°C)	15.3 ± 7.4	−4.4	9.4	16.4	21.8	28.2
RH (%)	80.3 ± 11.8	32.0	72.7	81.0	89.7	100.0
CO (mg/m^3^)	0.6 ± 0.2	0.3	0.5	0.6	0.7	1.9
NO_2_ (μg/m^3^)	22.5 ± 9.9	3.3	15.4	20.9	27.8	71.1
SO_2_ (μg/m^3^)	13.8 ± 14.4	3.0	6.0	8.9	15.4	159.0
PM_2.5_ (μg/m^3^)	32.4 ± 21.0	4.8	17.1	27.6	41.6	164.7
PM_10_ (μg/m^3^)	53.1 ± 30.8	8.1	30.7	45.7	67.6	263.3

Data exclude the period from December 18, 2022, to January 28, 2023, due to the COVID-19 outbreak.

### 3.2 Correlations between air pollutants and meteorological factors

Spearman correlation analysis revealed negative correlations between temperature, relative humidity and most pollutants, except for CO, which was positively correlated with relative humidity (*r* = 0.22). The correlation coefficient between temperature and SO_2_ was −0.60. Positive correlations were observed among air pollutants, with the strongest association between PM_2.5_ and PM_10_ (*r* = 0.95). Variance inflation factor analysis identified significant mutilcollinearity between PM_2.5_ and PM_10_ (VIF = 11.2), while CO, NO_2_, and SO_2_ exhibited no mutilcollinearity, with VIF values of 2.4, 3.6, and 2.8, respectively (Tables 3, 4).

**Table 3:** Correlation between air pollutants and meteorological factors (*r*).

Variables	Temp	RH	CO	NO_2_	SO_2_	PM_2.5_	PM_10_
Temp (°C)	1.00						
RH (%)	−0.34*	1.00					
CO (mg/m^3^)	−0.47*	0.22*	1.00				
NO_2_ (μg/m^3^)	−0.24*	−0.13*	0.65*	1.00			
SO_2_ (μg/m^3^)	−0.60*	−0.09*	0.69*	0.70*	1.00		
PM_2.5_ (μg/m^3^)	−0.38*	−0.25*	0.62*	0.72*	0.74*	1.00	
PM_10_ (μg/m^3^)	−0.21*	−0.42*	0.52*	0.75*	0.68*	0.95*	1.00

^*^p < 0.05.

**Table 4:** Variance inflation factor analysis.

Air pollutant	Variance inflation factor (VIF)
CO	2.4
NO_2_	3.6
SO_2_	2.8
PM_2.5_	11.2
PM_10_	11.2

A VIF > 10 indicates significant multicollinearity.

### 3.3 Lag effects of air pollutants on mortality

#### 3.3.1 Effects of SO_2_ on non-accidental mortality

**Figure 1:**
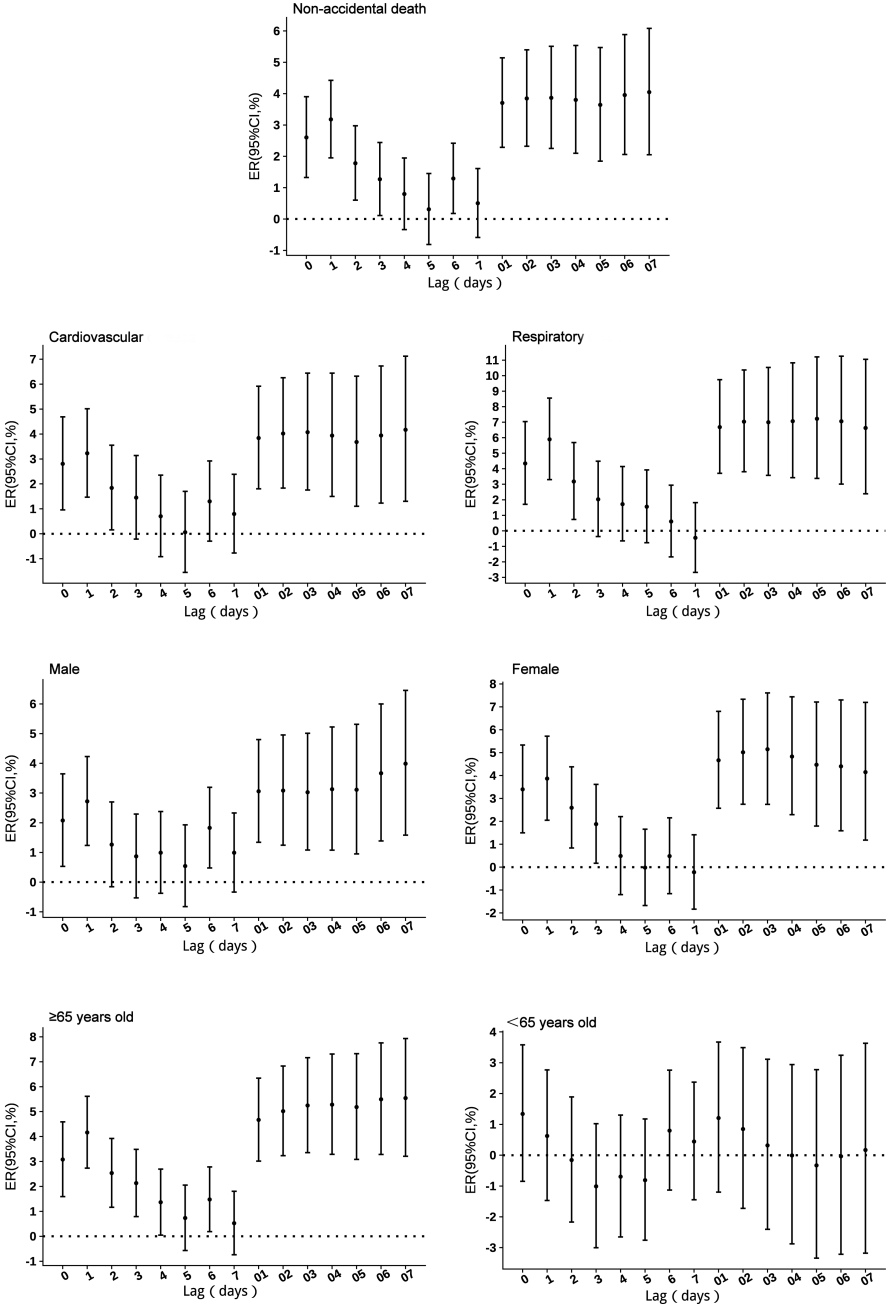
Excess risks of non-accidental mortality associated with SO_2_ exposure among residents in Guiyang.

No excess mortality risk from SO_2_ exposure was observed in individuals under 65 years. For other groups, the risks persisted for 1–3 days, peaking at lag 1. A 10 μg/m^3^ increase in SO_2_ concentration was associated with a 3.18% (95% CI: 1.95 to 4.42%) increase in non-accidental mortality risk, 3.23% (95% CI: 1.47 to 5.01%) in cardiovascular mortality risk, 5.89% (95% CI: 3.30 to 8.55%) in respiratory mortality risk. By sex, the risk increased by 2.72% (95% CI: 1.23 to 4.23%) for males, and 3.87% (95% CI: 2.05 to 5.72%) for females, while for individuals aged ≥65 years by 4.16% (95% CI: 2.73 to 5.61%). Cumulative lag effects showed no impact in the under 65 years group, while respiratory mortality and females risks peaked at lag 03 before gradually declining. Other groups exhibited increases at lags 06–07, with cumulative lag effects extending beyond 7 days (Figure 1).

#### 3.3.2 Effects of NO_2_ on non-accidental mortality

**Figure 2:**
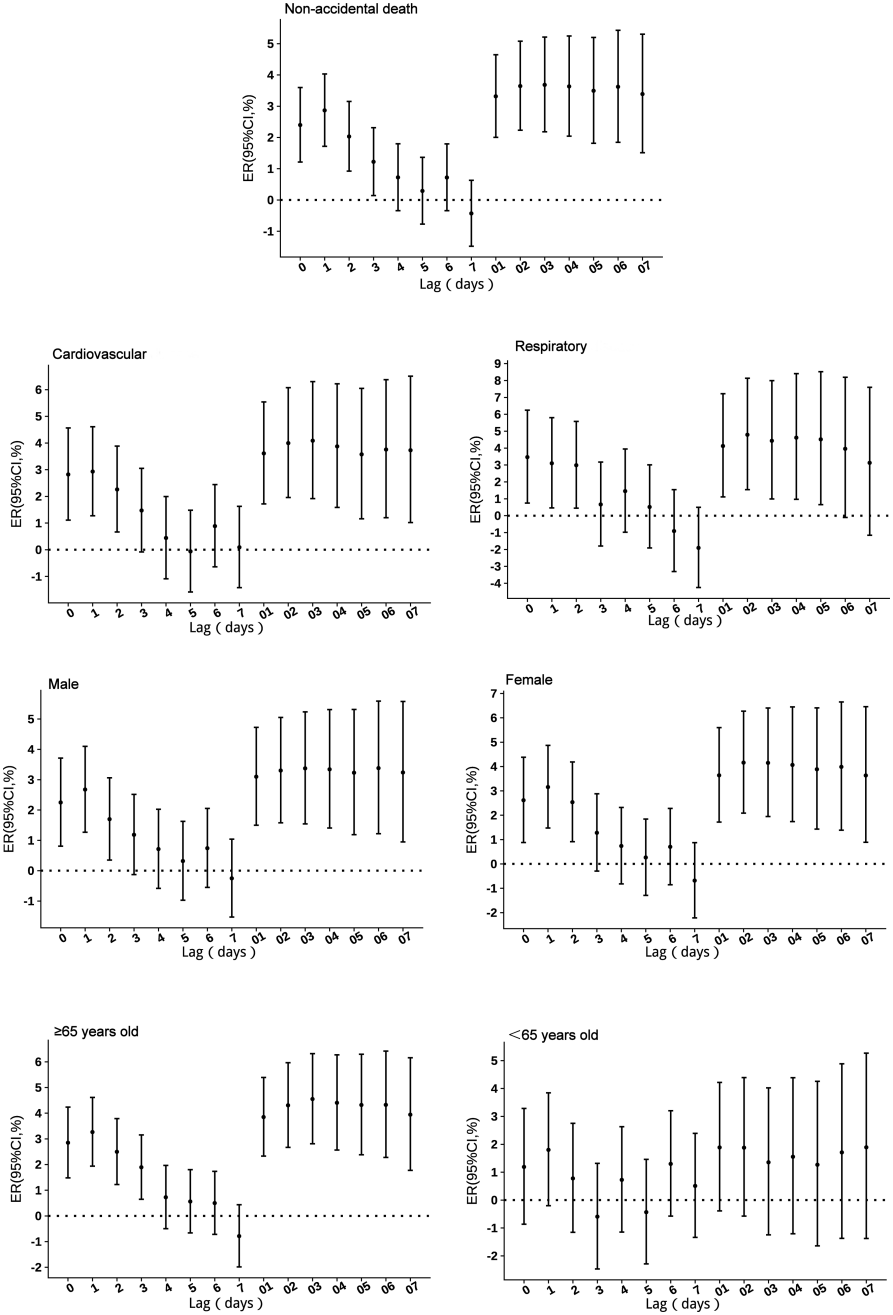
Excess risks of non-accidental mortality associated with NO_2_ exposure among residents in Guiyang.

No excess mortality risk from NO₂ exposure was observed in individuals under 65 years of age. For other groups, the risk persisted for 2–3 days. Respiratory mortality risk was highest on the day of exposure (lag 0), while other groups showed peak risk at lag 1. With a 10 μg/m^3^ increase in NO₂ concentration, non-accidental mortality risk increased by 2.87% (95% CI: 1.72 to 4.03%), respiratory mortality risk by 3.46% (95% CI: 0.75 to 6.25%), cardiovascular mortality risk by 2.93% (95% CI: 1.28 to 4.61%), male mortality risk by 2.67% (95% CI: 1.27 to 4.10%), female mortality risk by 3.16% (95% CI: 1.48 to 4.87%), and mortality risk in individuals aged ≥65 years by 3.27% (95% CI: 1.94 to 4.61%). Cumulative lag risks, except for the <65 years age group, peaked at lag 2–3 before gradually declining. The cumulative lag duration was 5 days for respiratory mortality risk and exceeded 7 days for all other groups (Figure 2).

#### 3.3.3 Effects of PM_2.5_ on non-accidental mortality

**Figure 3:**
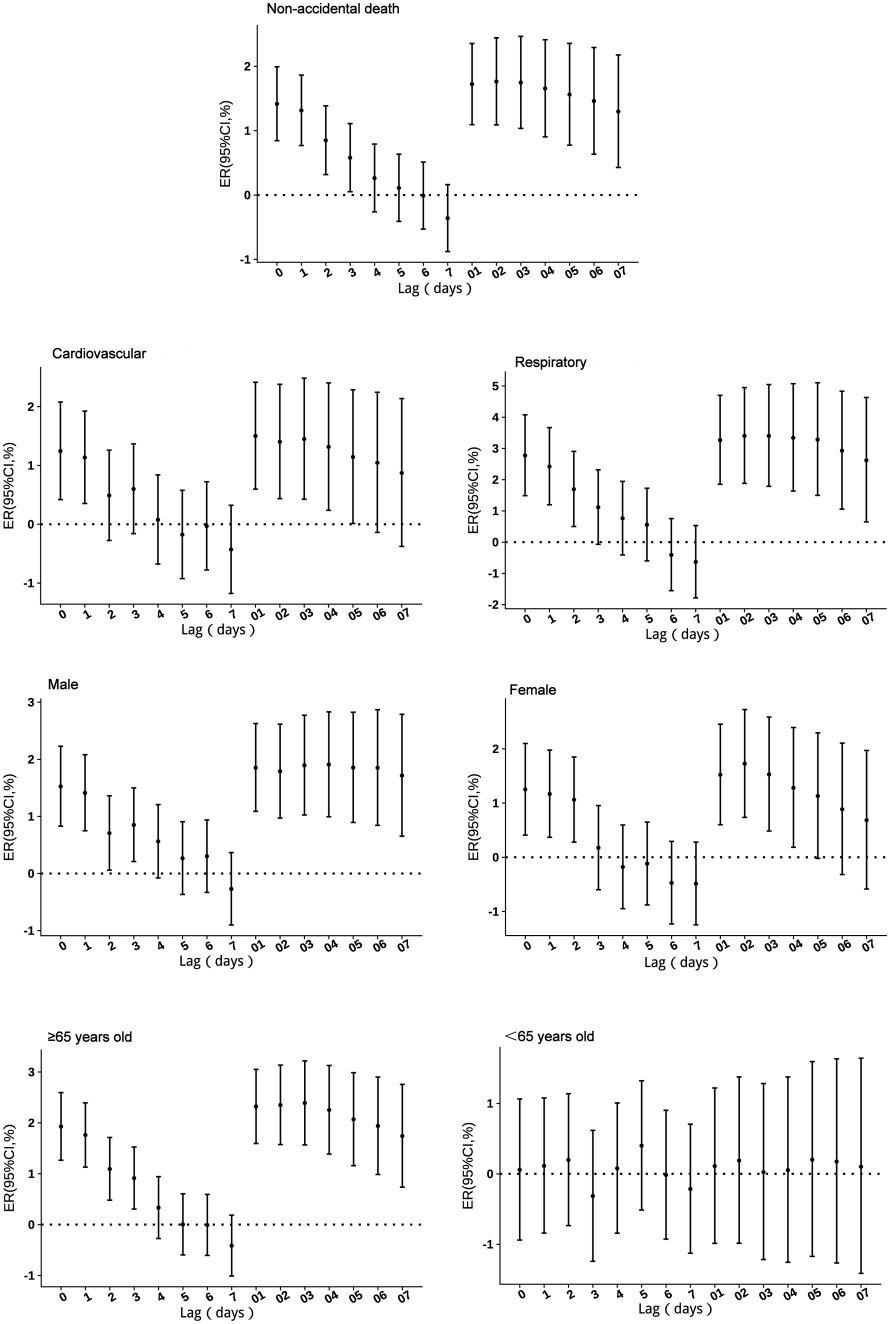
Excess risks of non-accidental mortality associated with PM_2.5_ exposure among residents in Guiyang.

No excess mortality risk from PM₂.₅ exposure was observed in individuals under 65 years of age. For other groups, single-day effects persisted for 1–3 days, with the highest risk occurring on the day of exposure (lag 0). With a 10 μg/m^3^ increase in PM_2.5_ concentration, non-accidental mortality risk increased by 1.42% (95% CI: 0.84 to 1.99%), cardiovascular mortality risk by 1.24% (95% CI: 0.42 to 2.08%), respiratory mortality risk by 2.78% (95% CI: 1.49 to 4.08%), male mortality risk by 2.72% (95% CI: 1.23 to 4.23%), female mortality risk by 1.25% (95% CI: 0.41 to 2.10%), and mortality risk in individuals aged ≥65 years by 1.93% (95% CI: 1.27 to 2.59%). Cumulative risks, except for the <65 years age group, peaked at lag 2 before gradually declining. The cumulative lag duration was 5 days for respiratory mortality risk, 4 days for female mortality risk, and exceeded 7 days for all other groups (Figure 3).

#### 3.3.4 Effects of PM_10_ on non-accidental mortality

**Figure 4:**
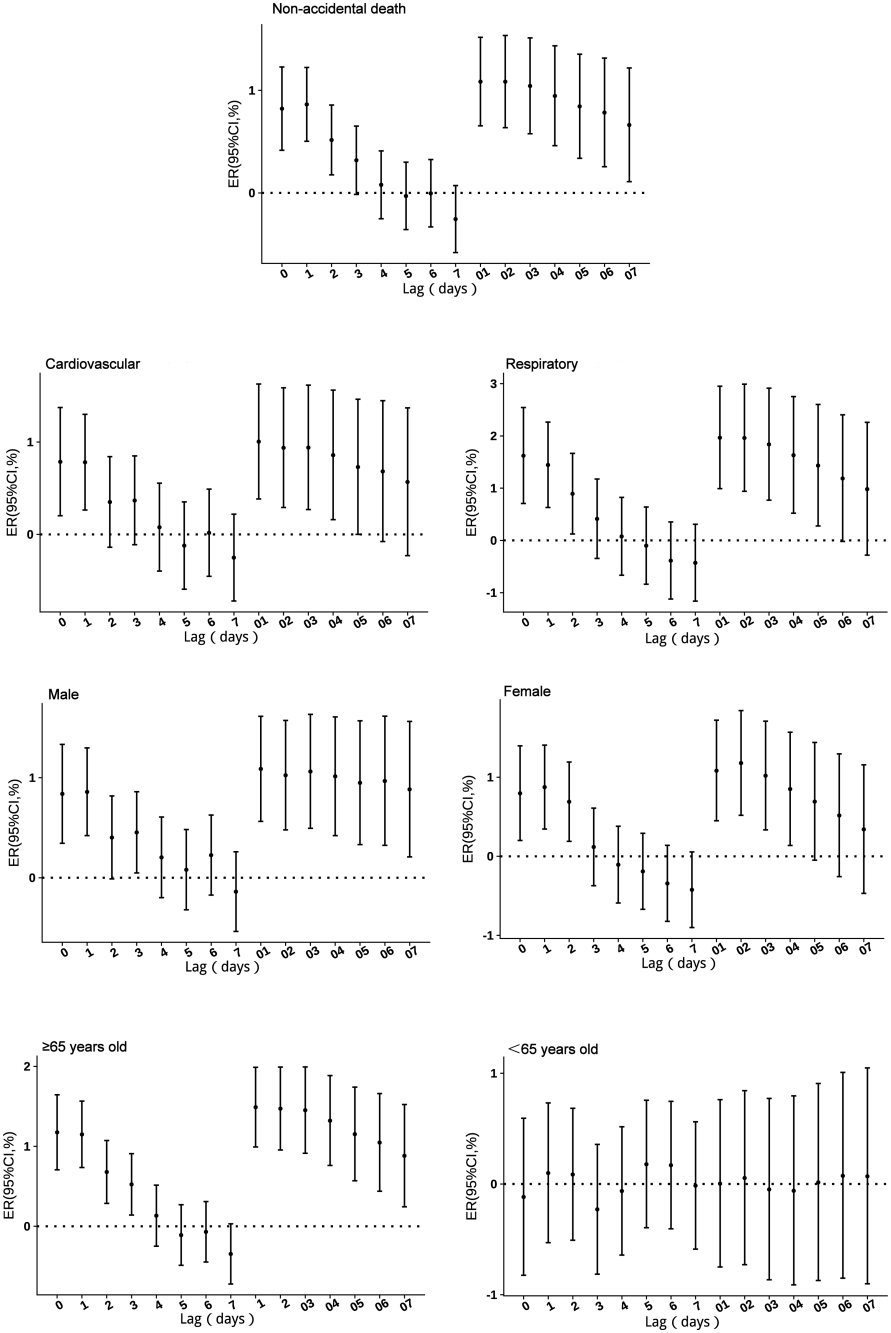
Excess risks of non-accidental mortality associated with PM_10_ exposure among residents in Guiyang.

No excess mortality risk from PM_10_ exposure was observed in individuals under 65 years of age. For other groups, single-day effects persisted for 1–3 days, with the highest risk occurring on the day of exposure (lag 0). With a 10 μg/m^3^ increase in PM_10_ concentration, non-accidental mortality risk increased by 0.86% (95% CI: 0.5 to 1.22%), cardiovascular mortality risk by 0.79% (95% CI: 0.2 to 1.37%), respiratory mortality risk by 1.62% (95% CI: 0.71 to 2.55%), male mortality risk by 0.86% (95% CI: 0.42 to 1.3%), female mortality risk by 0.87% (95% CI: 0.34 to 1.41%), and mortality risk in individuals aged ≥65 years by 1.17% (95% CI: 0.71 to 1.65%). Cumulative lag risks, except for the <65 years age group, peaked at lag 1–2 before gradually declining. The cumulative lag duration was 5 days for both cardiovascular and respiratory mortality risks, 4 days for female mortality risk, and exceeded 7 days for all other groups (Figure 4).

#### 3.3.5 Effects of CO on non-accidental mortality

**Figure 5:**
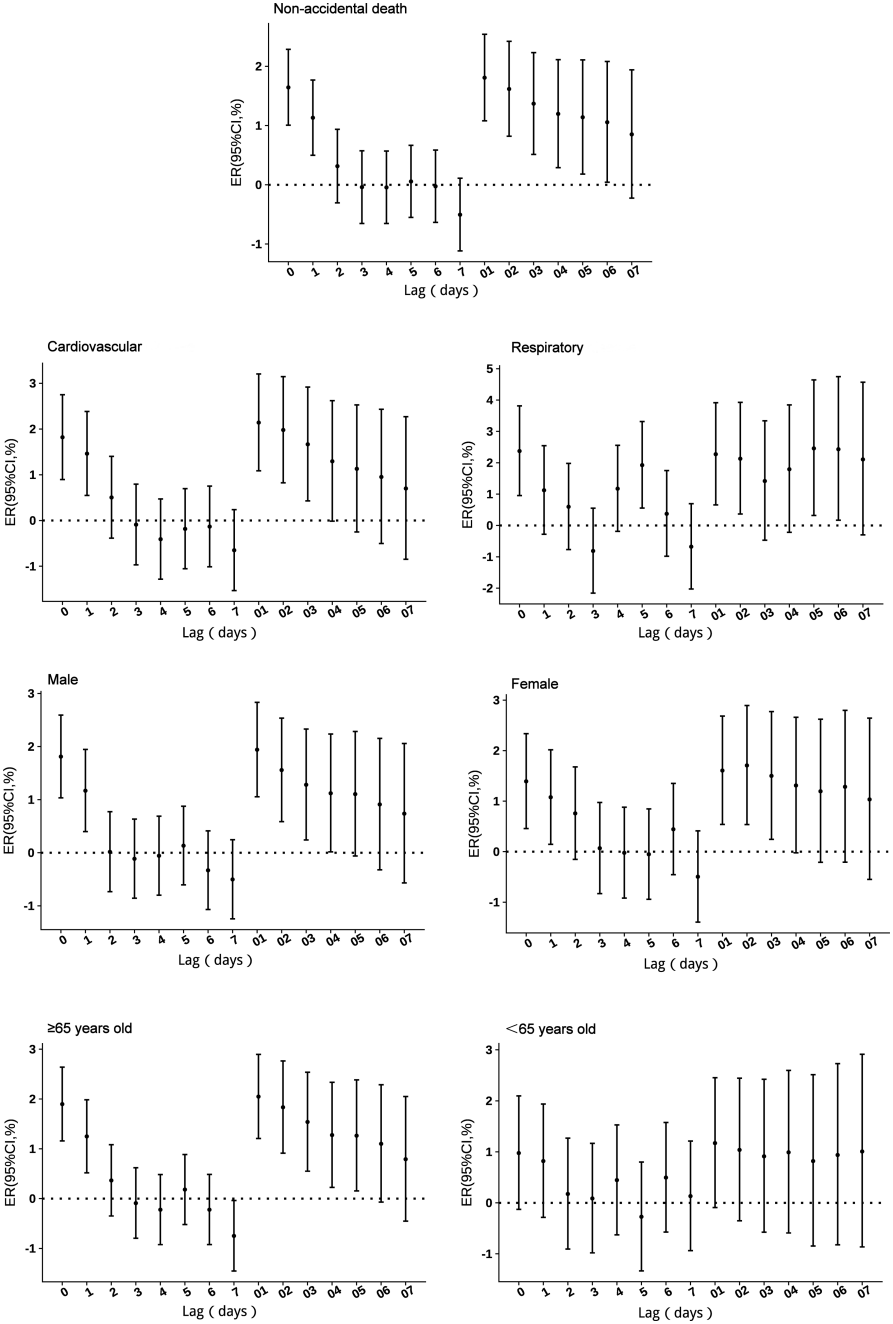
Excess risks of non-accidental mortality associated with CO exposure among residents in Guiyang.

No excess mortality risk from CO exposure was observed in individuals under 65 years of age. For other groups, single-day lag effects persisted for 0–1 days, with the highest risk occurring on the day of exposure (lag 0). With a 0.1 mg/m^3^ increase in CO concentration, non-accidental mortality risk increased by 1.64% (95% CI: 1.01 to 2.29%), cardiovascular mortality risk by 1.82% (95% CI: 0.89 to 2.75%), respiratory mortality risk by 2.38% (95% CI: 0.96 to 3.81%), male mortality risk by 1.81% (95% CI: 1.03 to 2.59%), female mortality risk by 1.39% (95% CI: 0.46 to 2.34%), and mortality risk in individuals aged ≥65 years by 1.90% (95% CI: 1.16 to 2.64%). Additionally, respiratory mortality risk showed an increase at lag 5. For cumulative lag risks, no effects were observed in the <65 years age group. In the respiratory mortality group, an increase in excess risk appeared at cumulative lag 5–6. For other groups, cumulative lag risks peaked at lag 1–2 before gradually declining, with cumulative effects persisting for 3–6 days (Figure 5).

#### 3.3.6 Excess mortality risks from air pollutants in cold and warm seasons

The study period was divided into cold and warm seasons for analysis. During the cold season, all pollutants demonstrated excess mortality risks across all groups, except for individuals under 65 years of age. For non-accidental diseases, mortality risk increased by 2.42% (95% CI: 1.05 to 3.80%) for SO_2_, 3.19% (95% CI: 1.72 to 4.67%) for NO_2_, 1.46% (95% CI: 0.79 to 2.13%) for PM_2.5_, 0.92% (95% CI: 0.44 to 1.41%) for PM_10_, and 1.47% (95% CI: 0.95 to 2.45%) for CO. No significant mortality risks were observed during the warm season (Table 5).

**Table 5:** Excess risks (%, 95CI) from air pollutants in cold and warm seasons.

Groups	SO_2_	NO_2_	PM_2.5_	PM_10_	CO
Cold season					
Non-accidental diseases	2.42% (1.05 to 3.80%)	3.19% (1.72 to 4.67%)	1.46% (0.79 to 2.13%)	0.92% (0.44 to 1.41%)	1.47% (0.95 to 2.45%)
Respiratory diseases	3.61% (0.79 to 6.51%)	4.13% (0.79 to 7.58%)	2.59% (1.10 to 4.10%)	1.45% (0.35 to 2.55%)	2.10% (0.44 to 3.79%)
Cardiovascular diseases	2.90% (0.93 to 4.91%)	3.52% (1.41 to 5.67%)	1.05% (0.10 to 2.02%)	0.75% (0.05 to 1.44%)	1.90% (0.82 to 2.99%)
Female	3.02% (1.04 to 5.05%)	3.15% (1.04 to 5.29%)	1.02% (0.06 to 1.99%)	0.70% (0.01 to 1.40%)	1.44% (0.36 to 2.53%)
Male	2.02% (0.36 to 3.71%)	3.22% (1.42 to 5.04%)	1.75% (0.94 to 2.57%)	1.07% (0.48 to 1.66%)	1.87% (0.95 to 2.79%)
≥65 years old	2.50% (0.94 to 4.08%)	3.78% (2.12 to 5.47%)	2.08% (1.33 to 2.84%)	1.31% (0.76 to 1.86%)	2.02% (1.17 to 2.88%)
<65 years old	2.18% (−0.20 to 4.61%)	1.54% (−1.03 to 4.17%)	−0.23% (−1.40 to 0.95%)	−0.13% (−0.98 to 0.74%)	0.82% (−0.49 to 2.14%)
Warm season					
Non-accidental diseases	1.71% (−2.92 to 6.56%)	0.13% (−1.82 to 2.13%)	0.93% (−0.21 to 2.08%)	0.33% (−0.41 to 1.08%)	0.83% (−0.44 to 2.12%)
Respiratory diseases	5.76% (−3.92 to 16.42%)	0.85% (−3.69 to 5.60%)	2.42% (−0.18 to 5.09%)	1.53% (−0.15 to 3.24%)	2.35% (−0.56 to 5.34%)
Cardiovascular diseases	−0.87% (−7.33 to 6.05%)	0.68% (−2.18 to 3.61%)	1.53% (−0.14 to 3.22%)	0.68% (−0.40 to 1.77%)	0.91% (−0.94 to 2.80%)
Female	1.97% (−5.02 to 9.46%)	0.69% (−2.23 to 3.70%)	1.41% (−0.30 to 3.16%)	0.68% (−0.44 to 1.81%)	0.72% (−1.18 to 2.66%)
Male	1.53% (−3.97 to 7.35%)	−0.24% (−2.59 to 2.18%)	0.61% (−0.77 to 2.00%)	0.10% (−0.79 to 1.00%)	0.91% (−0.63 to 2.48%)
≥65 years	3.87% (−1.71 to 9.77%)	0.00% (−2.29 to 2.34%)	0.90% (−0.43 to 2.25%)	0.48% (−0.39 to 1.36%)	0.69% (−0.80 to 2.20%)
<65 years	−3.38% (−10.65 to 4.47%)	0.46% (−2.91 to 3.94%)	0.95% (−1.01 to 2.95%)	−0.08% (−1.35 to 1.21%)	1.21% (−0.98 to 3.45%)

#### 3.3.7 Sensitivity analysis

The sensitivity analysis showed consistent results across different degrees of freedom (df) for time, humidity, and temperature, confirming the robustness of the model. For time (df = 6–8), humidity (df = 3–5), and temperature (df = 3–5), the excess mortality risks for SO_2_, NO_2_, PM_2.5_, PM_10_, and CO varied minimally, with only slight changes in effect estimates(Supplementary Table S3). In two-pollutant models, These findings indicate that the associations between air pollutants and mortality risks are stable under different model settings (Supplementary Table S4). The time-stratified sensitivity analysis revealed that the inclusion of the acute COVID-19 outbreak phase substantially altered the results (Supplementary Figure S1). The Baseline and Adjusted periods estimates remained broadly similar, whereas, whereas the results for the Full period differed substantially, particularly for CO, PM_10_, SO_2_, and NO_2_. Therefore, to mitigate this potential source of bias, the results from the full period analysis were excluded from our main analysis.

## 4 Discussion

This study provides a comprehensive analysis of the acute mortality effects of air pollution in Guiyang, China. Our approach utilized a time-series analysis, specifically employing a time-stratified case-crossover design with generalized additive models (GAMs), to examine these associations across strata of cause-specific mortality, sex, age, and season. The study covered 140,099 non-accidental deaths, with 59.44% occurring among males and 73.26% among those aged ≥65 years. The average temperature and relative humidity in Guiyang were 15.3°C and 80.3%, respectively. Daily average pollutant concentrations were relatively low compared to other Chinese cities, reflecting Guiyang’s minimal industrial activity. However, spearman correlation analysis revealed significant negative correlations between temperature and air pollutants during the study period, meaning cold season may be a dangerous factor.

Our analysis revealed that each pollutant induced mortality through distinct temporal patterns and biological mechanisms. A 10 μg/m^3^ increase in SO_2_ was associated with increases in non-accidental, respiratory, and cardiovascular mortality of 3.18% (95% CI: 1.95 to 4.42%), 5.89% (95% CI: 3.30 to 8.55%), and 3.23% (95% CI: 1.47 to 5.01%), respectively. As a water-soluble irritant gas, SO_2_ is rapidly absorbed by the upper respiratory tract, triggering vagus nerve reflexes that cause bronchial smooth muscle contraction, airway narrowing, and respiratory inflammation (15, 16). These mechanisms explain the stronger association with respiratory mortality. Additionally, SO_2_ may impact cardiovascular function through oxidative stress and interference with biological markers (17). Our findings align with studies linking short-term SO_2_ exposure to increased respiratory-related medical visits (18) and long-term exposure to bronchitis and reduced lung function.

NO_2_ exposure showed comparable acute health impacts, with single-day excess mortality risks peaking at lag 1 for most groups, except respiratory mortality, which was highest at lag 0. A 10 μg/m^3^ increase in NO_2_ concentration increased non-accidental mortality risk by 2.87% (95% CI: 1.72 to 4.03%), cardiovascular mortality by 2.93% (95% CI: 1.28 to 4.61%), and respiratory mortality by 3.46% (95% CI: 0.75 to 6.25%). These results are consistent with an Italian cohort study by Chiusolo et al. (19), which reported mortality increments of 2.09% (95% CI: 0.96 to 3.24%) for all-cause deaths, 3.48% (95% CI: 0.75 to 6.29%) for respiratory mortality and 2.63% (95% CI: 1.53 to 3.75%) for cardiovascular mortality per 10 μg/m^3^ increase in NO_2_. Cumulative lag effects persisted beyond 7 days for cardiovascular mortality, indicating more severe and prolonged damage compared to respiratory effects, which ceased after day 6. A meta-analysis supports greater cardiovascular damage from long-term NO_2_ exposure (20), with associations to coronary heart disease, stroke, and arrhythmia (21, 22).

Exposure to particulate matter was significantly associated with increased mortality, with the effects of PM_2.5_ being consistently stronger than those of PM_10_. A 10 μg/m^3^ increase in PM_2.5_ was associated with a 1.42% (95% CI: 0.84 to 1.99%) increase in non-accidental mortality, a 1.24% (95% CI: 0.42 to 2.08%) increase in cardiovascular mortality, and a 2.78% (95% CI: 1.49 to 4.08%) increase in respiratory mortality. In comparison, the same increase in PM_10_ resulted in lower excess mortality effects, with increases of 0.86% (95% CI: 0.50 to 1.22%) for non-accidental, 0.79% (95% CI: 0.20 to 1.37%) for cardiovascular, and 1.62% (95% CI: 0.71 to 2.55%) for respiratory mortality. The stronger health impact of PM_2.5_ aligns with its smaller particle size and higher reactivity, which enable deeper penetration into alveolar tissue and the absorption of toxic substances (23), resulting in both respiratory and cardiovascular damage as supported by prior research (24). Both pollutants exhibited similar acute effects, with single-day risks peaking at lag 0 and persisting for 1–3 days, while cumulative risks for both extended beyond 7 days for most groups. Furthermore, the strong correlation (r = 0.95) and significant multicollinearity (VIF = 11.2) observed between PM_2.5_ and PM₁₀ are noteworthy. This statistical relationship is expected, as PM_2.5_ is a physical subset of PM_10_. Additionally, the strong association suggests that both pollutants largely originate from common emission sources in Guiyang, such as vehicular traffic and coal combustion, highlighting the need for integrated control strategies (25).

CO exposure also presented a significant acute risk, with a 1.64% (95% CI: 1.01 to 2.29%) mortality increase per 0.1 mg/m^3^ rise, peaking at lag 0. This immediate effect is explained by its primary toxic mechanism: the formation of carboxyhemoglobin, which impairs the blood’s oxygen-carrying capacity and leads to systemic tissue hypoxia, primarily affecting cardiovascular and respiratory systems (26, 27).

Individuals aged ≥65 years exhibited higher vulnerability across all pollutants, with excess risks of 4.16% (95% CI: 2.73 to 5.61%) for SO_2_, 3.27% (95% CI: 1.94 to 4.61%) for NO_2_, 1.93% (95% CI: 1.27 to 2.59%) for PM_2.5_, 1.17% (95% CI: 0.71 to 1.65%) for PM_10_, and 1.90% (95% CI: 1.16 to 2.64%) for CO. This aligns with prior studies attributing increased susceptibility to compromised immune function and prevalent comorbidities in older adults (28, 29). Sex-stratified analyses revealed nuanced differences. Females showed greater susceptibility to SO_2_ with an excess risk of 3.87% (95% CI: 2.05 to 5.72%), an effect that persisted for multiple days and may be linked to higher airway reactivity and hormonal influences (30). In contrast, males were more vulnerable to PM_2.5_, showing an excess risk of 2.72% (95% CI: 1.23 to 4.23%) with extended cumulative effects, a finding possibly exacerbated by higher smoking rates which can amplify particulate matter toxicity (31). While risks for NO_2_, PM_10_, and CO were more comparable between sexes, these findings highlight how gender can modify susceptibility depending on the specific pollutant.

When dividing the study period into cold and warm seasons. All significant health risks were concentrated in the cold season, with no notable effects in the warm season, coinciding with higher pollutant concentrations during this period. This finding aligns with previous studies (32, 33). This pronounced seasonal difference is likely driven by two key factors: a surge in pollutant emissions from heating during winter, and adverse meteorological conditions to Guiyang. The city’s climate, heavily influenced by a quasi-stationary front, leads to humid, calm, and rainy winters that trap pollutants near ground level and result in sustained high-concentration exposures. The strong negative correlation between temperature and pollutant levels further supports the role of the cold season as the primary period of risk.

Several limitations should be noted in this study. First, as an ecological study, our analysis may be subject to ecological fallacy, meaning population-level associations may not accurately reflect individual exposure concentration-mortality relationships. Second, we were unable to control for important socioeconomic factors, particularly income level and occupation, which may influence exposure and vulnerability. Third, despite strict quality control, potential misdiagnosis or coding errors in mortality data cannot be ruled out. Fourth, the lack of indoor environmental data may introduce bias to our results. Despite these limitations, our findings hold significant public health relevance. Future research should aim to address these gaps by employing more detailed individual-level studies and enhanced data collection methods. Furthermore, conducting source apportionment analyses would help identify the specific pollution sources most strongly associated with these health effects, thereby providing more targeted evidence for effective policy interventions.

## 5 Conclusion

This study provides compelling evidence that short-term exposure to ambient air pollutants in Guiyang significantly increases the risk of non-accidental, cardiovascular, and respiratory mortality. The key public health message is that substantial health threats persist even at the city’s relatively moderate pollution levels, particularly for vulnerable segments of the population during specific high-risk periods. Our findings pinpoint a clear profile of this vulnerability: the older adults (aged ≥65 years) are consistently the most affected subgroup, facing elevated risks from all measured pollutants, while specific gender-based susceptibilities were observed for SO₂ and PM_2.5_. Based on these findings, we recommend targeted interventions during the cold season. Policymakers should consider reducing emissions from residential heating by encouraging the use of cleaner energy sources like natural gas or electric heating systems. Public health officials should also develop campaigns to promote protective measures for vulnerable groups, including the use of air purifiers and masks during high pollution periods.

## Data Availability

The data analyzed in this study is subject to the following licenses/restrictions: the datasets presented in this article are managed in strict accordance with the data security and confidentiality requirements imposed by the Guizhou Center for Disease Control and Prevention. Requests to access these datasets should be directed to gzcdc_gws@163.com.

## References

[1] Sorensen C Lehmann E Holder C Hu J Krishnan A Münzel T. Reducing the health impacts of ambient air pollution. BMJ. (2022) 379:e069487. doi: 10.1136/bmj-2021-069487 36223913

[2] Lee YG Lee PH Choi SM An MH Jang AS. Effects of air pollutants on airway diseases. Int J Environ Res Public Health. (2021) 18:9905. doi: 10.3390/ijerph18189905, PMID: 34574829 PMC8465980

[3] Hu X Knibbs LD Zhou Y Ou Y Dong GH Dong H. The role of lifestyle in the association between long-term ambient air pollution exposure and cardiovascular disease: a national cohort study in China. BMC Med. (2024) 22:93. doi: 10.1186/s12916-024-03316-z, PMID: 38439026 PMC10913402

[4] GBD 2021 Risk Factors Collaborators. Global burden and strength of evidence for 88 risk factors in 204 countries and 811 subnational locations, 1990-2021: a systematic analysis for the global burden of disease study 2021. Lancet. (2024) 403:2162–203. doi: 10.1016/S0140-6736(24)00933-4, PMID: 38762324 PMC11120204

[5] Xing YF Xu YH Shi MH Lian YX. The impact of PM2.5 on the human respiratory system. J Thorac Dis. (2016) 8:E69-74. doi: 10.3978/j.issn.2072-1439.2016.01.19, PMID: 26904255 PMC4740125

[6] Feng YM Thijs L Zhang ZY Bijnens EM Yang WY Wei FF. Glomerular function in relation to fine airborne particulate matter in a representative population sample. Sci Rep. (2021) 11:14646. doi: 10.1038/s41598-021-94136-1, PMID: 34282189 PMC8290004

[7] Hara A Sato T Kress S Suzuki K Pham KO Tajima A. Sex-specific associations between air pollutants and asthma prevalence in Japanese adults: a population-based study. Int J Environ Health Res. (2025) 35:310–8. doi: 10.1080/09603123.2024.2352597, PMID: 38741239

[8] Ethan CJ Mokoena KK Yu Y. Air pollution status in 10 mega-cities in China during the initial phase of the COVID-19 outbreak. Int J Environ Res Public Health. (2021) 18:3172. doi: 10.3390/ijerph18063172, PMID: 33808577 PMC8003380

[9] Cui L Zhou J Peng X Ruan S Zhang Y. Analyses of air pollution control measures and co-benefits in the heavily air-polluted Jinan city of China, 2013-2017. Sci Rep. (2020) 10:5423. doi: 10.1038/s41598-020-62475-0, PMID: 32214211 PMC7096483

[10] Tobias A Kim Y Madaniyazi L. Time-stratified case-crossover studies for aggregated data in environmental epidemiology: a tutorial. Int J Epidemiol. (2024) 53:dyae020. doi: 10.1093/ije/dyae020, PMID: 38380445 PMC10879751

[11] Janes H Sheppard L Lumley T. Case-crossover analyses of air pollution exposure data: referent selection strategies and their implications for bias. Epidemiology. (2005) 16:717–26. doi: 10.1097/01.ede.0000181315.18836.9d 16222160

[12] Lu Y Symons JM Geyh AS Zeger SL. An approach to checking case-crossover analyses based on equivalence with time-series methods. Epidemiology. (2008) 19:169–75. doi: 10.1097/EDE.0b013e3181632c24, PMID: 18223483

[13] Braga AL Zanobetti A Schwartz J. The lag structure between particulate air pollution and respiratory and cardiovascular deaths in 10 US cities. J Occup Environ Med. (2001) 43:927–33. doi: 10.1097/00043764-200111000-00001 11725331

[14] Liu C Chen R Sera F Vicedo-Cabrera AM Guo Y Tong S. Ambient particulate air pollution and daily mortality in 652 cities. N Engl J Med. (2019) 381:705–15. doi: 10.1056/NEJMoa1817364, PMID: 31433918 PMC7891185

[15] Barbone F Catelan D Pistelli R Accetta G Grechi D Rusconi F. A panel study on lung function and bronchial inflammation among children exposed to ambient SO₂ from an oil refinery. Int J Environ Res Public Health. (2019) 16:1057. doi: 10.3390/ijerph16061057, PMID: 30909566 PMC6466338

[16] Carlsen HK Valdimarsdóttir U Briem H Dominici F Finnbjornsdottir RG Jóhannsson T. Severe volcanic SO(2) exposure and respiratory morbidity in the Icelandic population - a register study. Environ Health. (2021) 20:23. doi: 10.1186/s12940-021-00698-y, PMID: 33639965 PMC7916308

[17] Hu Y Wu T Liu X Qiao D. Effects of exercise on the cardiovascular function of rats in a sulfur dioxide polluted environment. An Acad Bras Cienc. (2022) 94:e20211180. doi: 10.1590/0001-3765202220211180, PMID: 35674607

[18] Tomić-Spirić V Kovačević G Marinković J Janković J Ćirković A Đerić AM. Sulfur dioxide and exacerbation of allergic respiratory diseases: a time-stratified case-crossover study. J Res Med Sci. (2021) 26:109. doi: 10.4103/jrms.JRMS_6_20, PMID: 35126572 PMC8765521

[19] Chiusolo M Cadum E Stafoggia M Galassi C Berti G Faustini A. Short-term effects of nitrogen dioxide on mortality and susceptibility factors in 10 Italian cities: the EpiAir study. Environ Health Perspect. (2011) 119:1233–8. doi: 10.1289/ehp.1002904, PMID: 21586369 PMC3230391

[20] Chen X Qi L Li S Duan X. Long-term NO(2) exposure and mortality: a comprehensive meta-analysis. Environ Pollut. (2024) 341:122971. doi: 10.1016/j.envpol.2023.122971, PMID: 37984474

[21] Dong TF Zha ZQ Sun L Liu L-L Li X-Y Wang Y. Ambient nitrogen dioxide and cardiovascular diseases in rural regions: a time-series analyses using data from the new rural cooperative medical scheme in Fuyang, East China. Environ Sci Pollut Res Int. (2023) 30:51412–21. doi: 10.1007/s11356-023-25922-9, PMID: 36809617

[22] Wang Y Qu S Li T Chen L Yang L. Association between ambient air pollution and outpatient visits of cardiovascular diseases in Zibo, China: a time series analysis. Front Public Health. (2025) 12:1492056. doi: 10.3389/fpubh.2024.1492056, PMID: 39845652 PMC11750768

[23] Grzywa-Celińska A Krusiński A Milanowski J. 'Smoging kills' - effects of air pollution on human respiratory system. Ann Agric Environ Med. (2020) 27:1–5. doi: 10.26444/aaem/110477, PMID: 32208572

[24] Shin HH Parajuli RP Gogna P Maquiling A Dehghani P. Pollutant-sex specific differences in respiratory hospitalization and mortality risk attributable to short-term exposure to ambient air pollution. Sci Total Environ. (2021) 755:143135. doi: 10.1016/j.scitotenv.2020.143135, PMID: 33168238

[25] Li Y Chen Q Zhao H Wang L Tao R. Variations in PM10, PM2.5 and PM1.0 in an urban area of the Sichuan Basin and their relation to meteorological factors. Atmos. (2015) 6:150–63. doi: 10.3390/atmos6010150

[26] Gaalema DE. Carbon monoxide and its effects on those with cardiovascular disease. J Cardiopulm Rehabil Prev. (2022) 42:E55–6. doi: 10.1097/HCR.0000000000000733, PMID: 36044764 PMC9450230

[27] You J Liu Y Dong J Wang J Bao H. Ambient carbon monoxide and the risk of cardiovascular disease emergency room visits: a time-series study in Lanzhou, China. Environ Geochem Health. (2023) 45:7621–36. doi: 10.1007/s10653-023-01653-1 37395909

[28] Jin T Di Q RéQUIA WJ Danesh Yazdi M Castro E Ma T. Associations between long-term air pollution exposure and the incidence of cardiovascular diseases among American older adults. Environ Int. (2022) 170:107594. doi: 10.1016/j.envint.2022.107594, PMID: 36283157 PMC9798657

[29] Ju K Lu L Chen T Duan Z Chen D Liao W. Does long-term exposure to air pollution impair physical and mental health in the middle-aged and older adults? A causal empirical analysis based on a longitudinal nationwide cohort in China. Sci Total Environ. (2022) 827:154312. doi: 10.1016/j.scitotenv.2022.154312, PMID: 35248644

[30] Clougherty JE. A growing role for gender analysis in air pollution epidemiology. Ciênc Saúde Colet. (2011) 16:2221–38. doi: 10.1590/s1413-81232011000400021, PMID: 21584463

[31] Tsai HH Tantoh DM Lu WY Chen CY Liaw YP. Cigarette smoking and PM(2.5) might jointly exacerbate the risk of metabolic syndrome. Front Public Health. (2023) 11:1234799. doi: 10.3389/fpubh.2023.1234799 38288423 PMC10822970

[32] Wine O Osornio Vargas A Campbell SM Campbell S Hosseini V Koch C. Cold climate impact on air-pollution-related health outcomes: a scoping review. Int J Environ Res Public Health. (2022) 19:1473. doi: 10.3390/ijerph19031473, PMID: 35162495 PMC8835073

[33] Yu LJ Li XL Wang YH Zhang HY Ruan SM Jiang BG. Short-term exposure to ambient air pollution and influenza: a multicity study in China. Environ Health Perspect. (2023) 131:127010. doi: 10.1289/EHP12146, PMID: 38078423 PMC10711743

