# Supplementary material for: Association between air pollution and non-accidental mortality in Guiyang, China: a time-series analysis (2013–2023)
Source: Front Public Health. 2025 Jul 16;13:1602900. doi: 10.3389/fpubh.2025.1602900 (PMC12307342; doi:10.3389/fpubh.2025.1602900)
Supplement: Supplementary file 1 [file Data_Sheet_1.docx]

**TABLE S1** Distributions of non-accidental mortality in Guiyang, 2013-2023

| Variables | n | x±s | Min | P25 | P50 | P75 | Max |
| --- | --- | --- | --- | --- | --- | --- | --- |
| Number of non-accidental deaths | 147262 | 36.7±22.8 | 1.0 | 21.0 | 37.0 | 48.0 | 367.0 |
| Number of respiratory system deaths | 23568 | 5.9±7.3 | 0.0 | 3.0 | 5.0 | 7.0 | 132.0 |
| Number of circulatory system deaths | 62424 | 15.5±9.6 | 0.0 | 9.0 | 15.0 | 21.0 | 135.0 |
| Male | 87817 | 21.9±14.3 | 0.0 | 13.0 | 21.0 | 28.0 | 224.0 |
| Female | 59445 | 14.8±9.5 | 0.0 | 8.0 | 15.0 | 20.0 | 143.0 |
| Age 0-64 years | 38422 | 9.6±5.0 | 0.0 | 6.0 | 9.0 | 13.0 | 41.0 |
| Age ≥ 65 years | 108840 | 27.1±19.4 | 0.0 | 15.0 | 27.0 | 36.0 | 327.0 |

**TABLE S2** Daily air pollutants concentration and meteorological factors in Guiyang, 2013-2023

| Variables | x±s | Min | *P*25 | *P*50 | *P*75 | Max |
| --- | --- | --- | --- | --- | --- | --- |
| Temp(℃) | 15.2±7.4 | -4.4 | 9.3 | 16.2 | 21.7 | 28.2 |
| RH(%) | 80.2±11.8 | 27.0 | 72.5 | 81.0 | 89.7 | 100.0 |
| CO (mg/m^3^) | 0.6±0.2 | 0.3 | 0.5 | 0.6 | 0.7 | 1.9 |
| NO_2_(μg/m^3^) | 22.5±9.9 | 3.3 | 15.3 | 20.8 | 27.8 | 71.1 |
| SO_2_ (μg/m^3^) | 13.8±14.3 | 3.0 | 6.0 | 8.9 | 15.4 | 159.0 |
| PM_2.5_(μg/m^3^) | 32.5±21.0 | 4.8 | 17.2 | 27.7 | 41.7 | 164.8 |
| PM_10_(μg/m^3^) | 53.2±30.7 | 8.1 | 30.8 | 45.8 | 67.7 | 263.3 |

**TABLE S3** Sensitivity analysis of excess risks (%, 95%CI) associated with air pollutants under varying degrees of freedom (df) for time, humidity, and temperature.

| Variables | SO_2_ | NO_2_ | PM_2.5_ | PM_10_ | CO |
| --- | --- | --- | --- | --- | --- |
| df for time |  |  |  |  |  |
| 6 | 2.604% (1.324% to 3.901%) | 2.400% (1.215% to 3.598%) | 1.417% (0.844% to 1.994%) | 0.821% (0.416% to 1.228%) | 1.644% (1.007% to 2.286%) |
| 7 | 2.604% (1.324% to 3.901%) | 2.400% (1.215% to 3.598%) | 1.417% (0.844% to 1.994%) | 0.821% (0.416% to 1.228%) | 1.644% (1.007% to 2.286%) |
| 8 | 2.604% (1.324% to 3.901%) | 2.400% (1.215% to 3.598%) | 1.417% (0.844% to 1.994%) | 0.821% (0.416% to 1.228%) | 1.644% (1.007% to 2.286%) |
| df for humidity |  |  |  |  |  |
| 3 | 2.604% (1.324% to 3.901%) | 2.400% (1.215% to 3.598%) | 1.417% (0.844% to 1.994%) | 0.821% (0.416% to 1.228%) | 1.644% (1.007% to 2.286%) |
| 4 | 2.604% (1.324% to 3.901%) | 2.400% (1.215% to 3.598%) | 1.417% (0.844% to 1.994%) | 0.821% (0.416% to 1.228%) | 1.644% (1.007% to 2.286%) |
| 5 | 2.604% (1.324% to 3.901%) | 2.400% (1.215% to 3.598%) | 1.417% (0.844% to 1.994%) | 0.821% (0.416% to 1.228%) | 1.644% (1.007% to 2.286%) |
| df for temperature |  |  |  |  |  |
| 3 | 2.604% (1.324% to 3.901%) | 2.400% (1.215% to 3.598%) | 1.417% (0.844% to 1.994%) | 0.821% (0.416% to 1.228%) | 1.644% (1.007% to 2.286%) |
| 4 | 2.605% (1.325% to 3.902%) | 2.405% (1.220% to 3.603%) | 1.420% (0.847% to 1.996%) | 0.823% (0.417% to 1.230%) | 1.643% (1.006% to 2.285%) |
| 5 | 2.605% (1.325% to 3.902%) | 2.410% (1.225% to 3.608%) | 1.422% (0.849% to 1.999%) | 0.825% (0.419% to 1.232%) | 1.644% (1.007% to 2.286%) |

**TABLE S4** Sensitivity analysis of excess risks (%, 95%CI) in two-pollutant model

| Pollutant | ER (%, 95%CI) |
| --- | --- |
| SO_2_+ |  |
| NO_2_ | 1.696%（0.196% to 3.219%） |
| PM_2.5_ | 1.401%（-0.042% to 2.866%） |
| PM_10_ | 1.739%（0.271% to 3.228%） |
| CO | 1.379%（-0.041% to 2.820%） |
| NO_2_+ |  |
| PM_2.5_ | 1.178%（-0.198% to 2.574%） |
| PM_10_ | 1.494%（0.014% to 2.995%） |
| CO | 0.971%（-0.448% to 2.410%） |
| PM_2.5_+ |  |
| PM_10_ | -0.693%（-1.728% to 0.354%） |
| CO | 1.153%（0.431% to 1.880%） |
| PM_10_+ |  |
| CO | 1.330%（0.607% to 2.059%） |

| **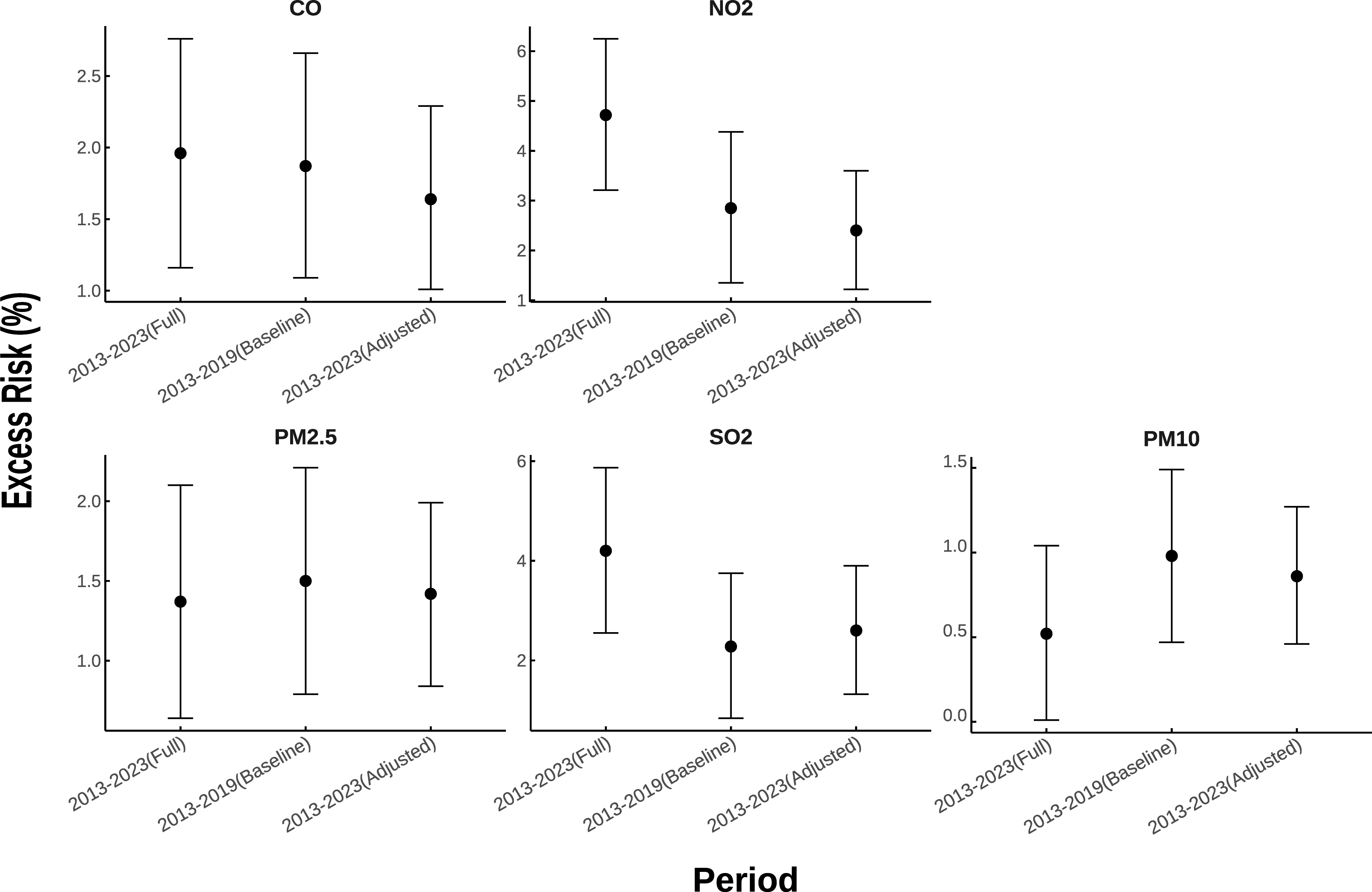** |
| --- |
| **FIGURE S1** Pollutant-Related Excess Mortality Risk Across Time Periods (2013–2023)  *Note：Full: 2013–2023 including all available data. Baseline: 2013–2019 (pre-pandemic period); Adjusted: 2013–2023 excluding the acute outbreak period (Dec 18, 2022–Jan 28, 2023);* |
